# Metformin Attenuates Testosterone-Induced Prostatic Hyperplasia in Rats: A Pharmacological Perspective

**DOI:** 10.1038/srep15639

**Published:** 2015-10-23

**Authors:** Hala H. Mosli, Ahmed Esmat, Reem T. Atawia, Sherif M. Shoieb, Hisham A. Mosli, Ashraf B. Abdel-Naim

**Affiliations:** 1 Department of Internal Medicine (Endocrinology), King Abdulaziz University, Jeddah, Saudi Arabia; 2 Department of Pharmacology and Toxicology, Faculty of Pharmacy, Ain Shams University, Cairo, Egypt; 3 Department of Urology, Faculty of Medicine, King Abdulaziz University, Jeddah, Saudi Arabia; 4 Department of Pharmacology and Toxicology, Faculty of Pharmacy, King Abdulaziz University, Jeddah, Saudi Arabia

## Abstract

Benign prostatic hyperplasia (BPH) is uncontrolled proliferation of prostate tissue. Metformin, a widely prescribed anti-diabetic agent, possesses anticancer activity through induction of apoptotic signaling and cell cycle arrest. This study aimed to investigate the protective effect of metformin against experimentally-induced BPH in rats. Treatment with 500 and 1000 mg/kg metformin orally for 14 days significantly inhibited testosterone-mediated increase in the prostate weight & prostate index (prostate weight/body weight [mg/g]) and attenuated the pathological alterations induced by testosterone. Mechanistically, metformin significantly protected against testosterone-induced elevation of estrogen receptor-α (ER-α) and decrease of estrogen receptor-β (ER-β) expression, with no significant effect of androgen receptor (AR) and 5α-reductase expression. It decreased mRNA expression of IGF-1 and IGF-1R and protein expression ratio of pAkt/total Akt induced by testosterone. Furthermore, it significantly ameliorated testosterone–induced reduction of mRNA expression Bax/Bcl-2 ratio, P21 and phosphatase and tensin homolog (PTEN) and AMPK [PT-172] activity. In conclusion, these findings elucidate the effectiveness of metformin in preventing testosterone-induced BPH in rats. These results could be attributed, at least partly, to its ability to enhance expression ratio of ER-β/ER-α, decrease IGF-1, IGF-1R and pAkt expressions, increase P21, PTEN, Bax/Bcl-2 expressions and activate AMPK with a subsequent inhibition of prostate proliferation.

Benign prostatic hyperplasia (BPH) is one of the most serious urinary system disorders in elderly men and characterized by hyperplasia of prostatic tissues^1^. Although androgenic signaling represents the primary stimuli for prostatic proliferation and BPH development, several lines of evidence suggested the implication of estrogen action via its distinct receptors; estrogen receptor-α (ER-α) and estrogen receptor-β (ER-β)^2^. ER-β regulates cellular growth and promotes apoptotic signaling pathways in the prostate. Whereas, ER-α promote cellular proliferation and enhance survival and mitogenic pathway^3^.

Several growth factors contribute to BPH progression particularly insulin growth factor-1 (IGF-1) action via its receptor IGF-1R has been shown to promote prostatic growth and development via activation of phosphoinositol-3-kinase (PI3K)/protein kinase B (PKB/Akt)^4^. In addition, deregulation in the prostatic IGF system has been previously documented in BPH patients^5^. Phosphatase and tensin homologue deleted on chromosome 10 (PTEN), a tumor suppressor gene whose expression is reduced in several tumors. PTEN has been shown to suppress IGF1-induced Akt phosphorylation and in turn cell proliferation *in-vitro*^6^.

Metformin is an oral anti-hyperglycemic agent of biguanide class and insulin sensitizer which inhibits hepatic gluconeogenesis and enhance peripheral glucose uptake^7^. It also reduces cancer risk in diabetic patients^8^ and possesses antineoplastic activity against several tumors including the prostate^9^, ovarian^10^, breast^11^ and endometrial carcinoma models through induction of apoptotic signaling and cell cycle arrest^12^. The antineoplastic and antihyperglycemic effects of metformin would be attributed to its beneficial adenosine monophosphate-activated protein kinase (AMPK) activation ability^13^. Adenosine monophosphate-activated protein kinase (AMPK) is a heteromeric serine/threonine kinase that serves to regulate energy homeostasis^14^. AMPK provides inhibition of several pro-oncogenic pathways and indirectly inhibit IGF-1/IGF-1R signaling^15^. The aim of this study was to investigate the protective effect of metformin against experimentally-induced prostatic hyperplasia in rats with emphasis on its potential effects on certain key players of IGF-1/IGF-1R signaling pathway.

## Results

### Prostate weight and prostate index

**Figure 1:**
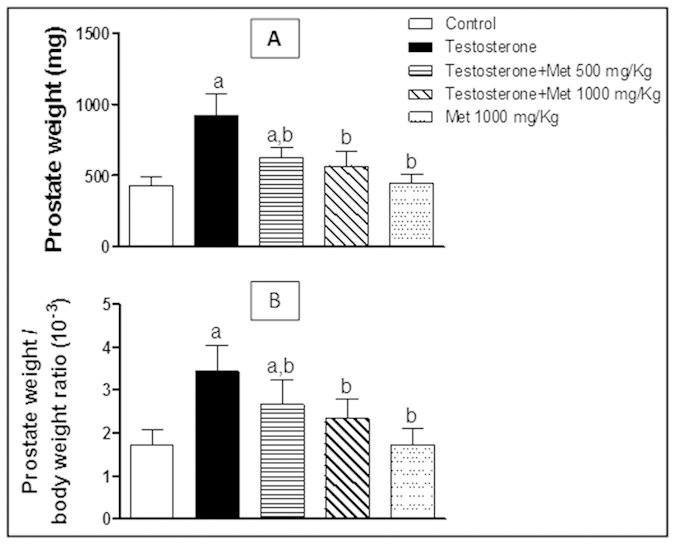
Effect of metformin treatment in two different doses (500 mg/kg, 1000 mg/kg, orally, for 2 weeks) on the prostate weight (A), prostate weight/body weight (B). Data are represented by mean ± SD (*n* = 8). a or b, Statistically significant from control or testosterone-only treated group respectively at p < 0.05 using one-way ANOVA followed by Tukey–Kramer as a post hoc test.

Animals treated with testosterone (3 mg/kg/day, 5 days /week for 2 weeks, S.C.) showed a significant increase in prostate weight and prostate weight/body weight ratio by 115.03% and 99.2% respectively compared to the control group. In comparison with the testosterone-only treated group, metformin (500 and 1000 mg/kg/day, 5 days/week for 2 weeks, p.o., given concomitantly with testosterone) significantly decreased the prostate weight gain induced with testosterone by 32.31% and 38.75% respectively, and decreased the prostate weight/body weight ratio by 22.69% and 32.08% respectively. Metformin alone (1000 mg/kg p.o.) did not show any significant difference from the control group (Fig. 1A,B).

### Histopathological examination

**Figure 2:**
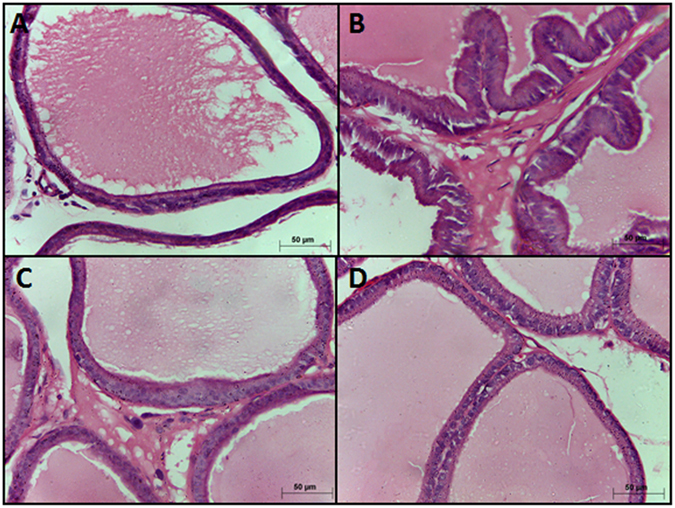
Histological examination of hematoxylin-eosin sections of rat ventral prostates (×40). (**A**) Section taken from the prostate of the control group shows normal morphological structure of the lining epithelial cells. (**B**) Section taken from the prostate of testosterone only treated group exhibit hypertrophy with increased epithelial thickness and polyps formations. (**C**,**D**) Sections taken from the prostate of testosterone groups co-treated with 500 mg/kg (**C**) or 1000 mg/kg (**D**) metformin show marked reduction in prostate hypertrophy and hyperplasia induced by testosterone.

Sections from control group showed normal histological architecture of the prostate (Fig. 2A). Testosterone–treated group showed luminal epithelial hyperplasia with intraluminal polyps as well as engorged blood vessels (Fig. 2B). Co-treatment with metformin 500 and 1000 mg/kg attenuated the pathological alterations induced by testosterone (Fig. 2C,D).

### Assessment of ER-α and ER-β mRNA expression

**Figure 3:**
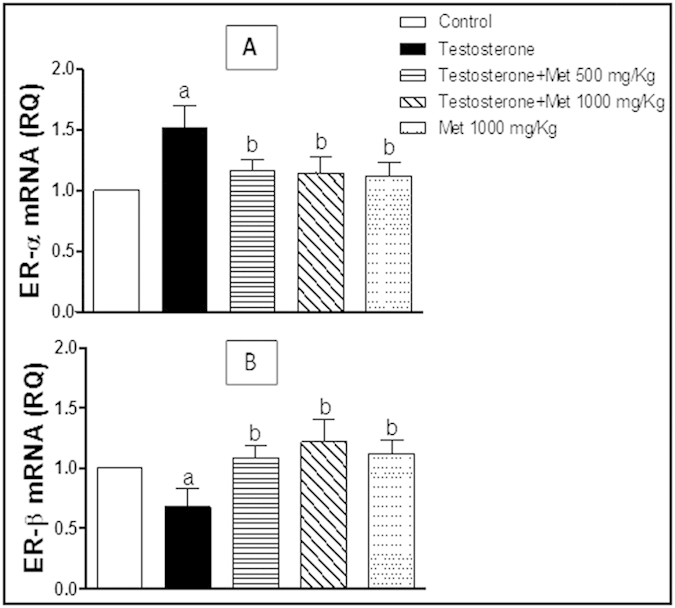
Quantitative RT-PCR of ER-α (A) and ER-β(B) mRNA expression expressed as relative quantification (RQ) compared to the control group which was assigned a value of 1. Each bar represents mean ± SD for a group of 3 rats. a or b, Statistically significant from the control or testosterone–only treated group, respectively, p < 0.05 using one-way ANOVA followed by Tukey–Kramer as a post-hoc test.

Testosterone administration induced a significant increase in mRNA expression of ER-α (Fig. 3A) while significantly decreased that of ER-β (Fig. 3B). Co-administration of metformin (500 and 1000 mg/kg, p.o.) significantly protected against this testosterone-mediated elevation of mRNA expression of ER-α (Fig. 3A) and significantly protected against the testosterone-induced decrease in mRNA expression of ER-β (Fig. 3B). With metformin alone (1000 mg/kg p.o.), the expressions of ER-α and ER-β and were not significantly different from the control group (Fig. 3A,B).

### Assessment of androgen receptor (AR) and 5α-redctase II mRNA expression

**Figure 4:**
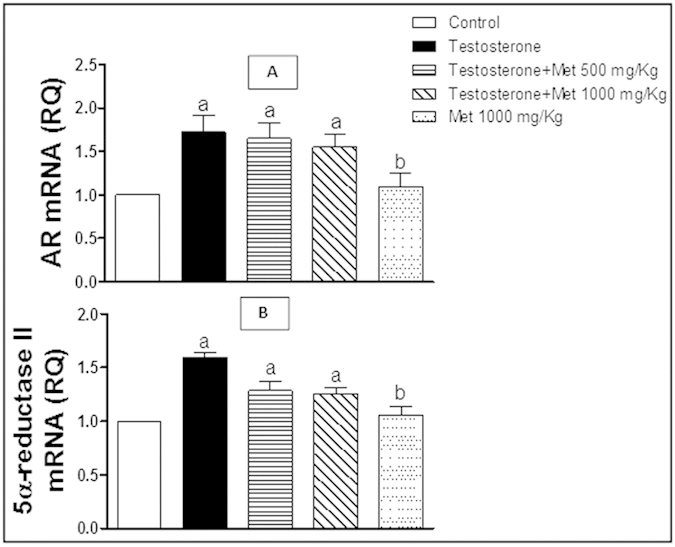
Quantitative RT-PCR of AR (A) and 5α-reductase II (B) mRNA expression expressed as relative quantification (RQ) compared to the control group which was assigned a value of 1. Each bar represents mean ± SD for a group of 3 rats. a or b, Statistically significant from the control or testosterone–only treated group, respectively, p < 0.05 using one-way ANOVA followed by Tukey–Kramer as a post-hoc test.

Testosterone administration significantly increased mRNA expression of both AR and 5α-redctase II (Fig. 4A,B), compared to the control group. Co-administration of metformin (500 and 1000 mg/kg, p.o.) reduced this testosterone-mediated elevation but failed to show any statistical difference from the testosterone-treated group (Fig. 4A,B). Metformin alone (1000 mg/kg p.o.) did not show any significant difference from the control group (Fig. 4A,B).

### Assessment of IGF-1, IGF-1R, PTEN, P21, Bax and Bcl-2 mRNA expression

**Figure 5:**
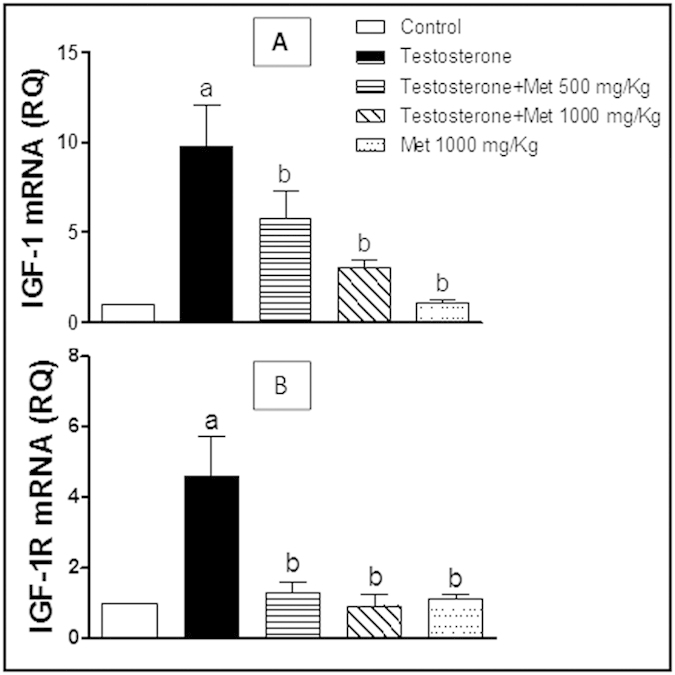
Quantitative RT-PCR of IGF-1 (A) and IGF-1R (B) mRNA expression expressed as relative quantification (RQ) compared to the control group which was assigned a value of 1. Each bar represents mean ± SD for a group of 3 rats. a or b, Statistically significant from the control or testosterone–only treated group, respectively, p < 0.05 using one-way ANOVA followed by Tukey–Kramer as a post-hoc test.

**Figure 6:**
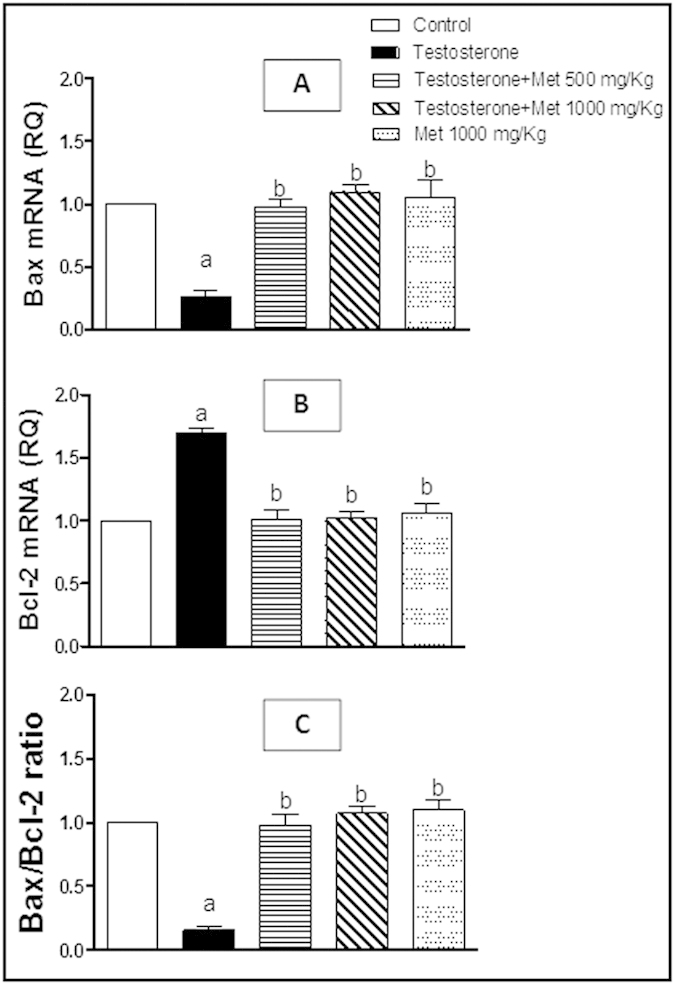
Quantitative RT-PCR of Bax (A) and Bcl-2 (B) mRNA expression expressed as relative quantification (RQ) compared to the control group which was assigned a value of 1. (C) the ratio of Bax to Bcl-xl after metformin administration in testosterone-treated/untreated groups. Each bar represents mean ± SD for a group of 3 rats. a or b, Statistically significant from the control or testosterone–only treated group, respectively, p < 0.05 using one-way ANOVA followed by Tukey–Kramer as a post-hoc test.

**Figure 7:**
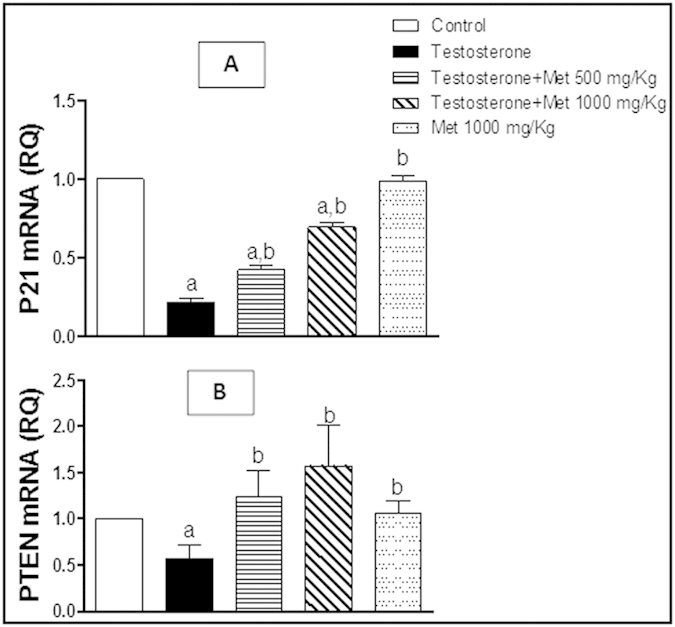
Quantitative RT-PCR of P21 (A) and PTEN (B) mRNA expression expressed as relative quantification (RQ) compared to the control group which was assigned a value of 1. Each bar represents mean ± SD for a group of 3 rats. a or b, Statistically significant from the control or testosterone–only treated group, respectively, p < 0.05 using one-way ANOVA followed by Tukey–Kramer as a post-hoc test.

The group of rats treated with testosterone-only showed significant up-regulation of IGF-1, IGF-1R (Fig. 5A,B) and Bcl-2 mRNA expression (Fig. 6B) while showed significant down-regulation of Bax mRNA expression (Fig. 6A), PTEN and P21 (Fig. 7A,B) and compared to the control group. Co-administration of metformin (500 and 1000 mg/kg, p.o.) significantly protected against the testosterone-mediated elevation of mRNA expression of IGF-1, IGF-1R (Fig. 5A,B) and Bcl-2 (Fig. 6B) and significantly protected against the testosterone-induced decrease in mRNA expression of Bax (Fig. 6A), P21and PTEN (Fig. 7A,B). Metformin alone (1000 mg/kg p.o.) did not show any significant difference in all these measured parameters, compared to the control group.

### Assessment of AMPKα [PT-172] concentration

**Figure 8:**
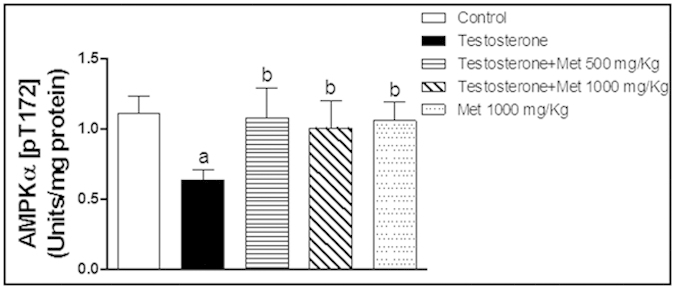
Effect of metformin treatment with (500 mg/kg, 1000 mg/kg, orally, for 2 weeks) on the prostatic concentration of phosphorylated AMPKα at Thr-172 expressed at (units/mg protein) in testosterone-treated and untreated rats. Data are expressed as mean ± SD (n = 8). a or b: Statistically significant from control or testosterone group, respectively at p < 0.05 using one-way ANOVA followed by Tukey–Kramer as a post hoc test.

As shown in Fig. 8, AMPKα [PT-172] concentration was significantly decreased in testosterone-treated rats as compared with control rats by 43% while co-treatment with metformin 500 mg/kg and 1000 mg/kg significantly ameliorated testosterone–induced depletion in AMPKα [PT-172] concentration by 70% and 59% respectively. Metformin alone (1000 mg/kg p.o.) did not show any significant difference from the control group.

### Assessment of pAkt/Total Akt ratio

**Figure 9:**
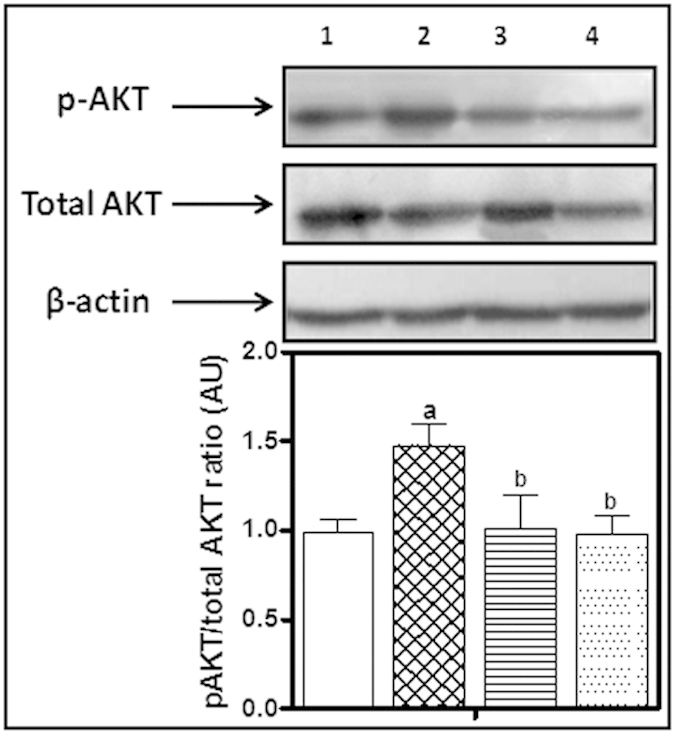
(**A**) Western blot analysis of pAKT& total AKTexpression in prostate tissues. (1) Untreated control, (2) Testosterone-induced BPH group (3 mg/kg, 5 days/week for 2 weeks, S.C.), (3) Testosterone-induced BPH treated with metformin (500 mg/kg, orally, for 2 weeks) (4) Testosterone-induced BPH treated with metformin (1000 mg/kg, orally, for 2 weeks). (**B**) Densitometric quantitation of pAKT/total AKT ratio. Data are expressed as mean ± SD (n = 3). a or b: Statistically significant from control or testosterone group, respectively at P < 0.05 using one-way ANOVA followed by Tukey–Kramer as a post hoc test.

The expression levels of pAkt and total Akt in prostate tissue following metformin treatment (500 & 1000 mg/kg orally for 2 weeks) was investigated by Western blot (Fig. 9). No significant change in the total protein expression of Akt was observed. However, obvious increase in pAkt expression was observed in testosterone-treated group compared to the corresponding control. Treatment with metformin at 500 and 1000 mg/kg decreased pAkt expression, as shown in (Fig. 9A). The densitometric quantitation of pAkt/total Akt ratio (Fig. 9B) indicates a significant increase in pAkt/total Akt ratio, compared to the control group. Metformin treatment significantly reduced this ratio as compared to testosterone-alone treated group with no significant difference from the control group.

## Discussion

BPH is considered the most common urological disorder affecting men above 50 years of age with a prevalence of up to 85% in older age groups^1^. It is characterized by non-cancerous enlargement and uncontrolled proliferation of smooth muscles and epithelial cells of the prostatic tissue. Clinically, BPH is manifested as LUTS with a significant negative impact on the quality of life^16^. The progression of BPH is dependent on several factors such as growth factors, adrenergic stimulation and inflammatory processes, which drive proliferative environment^17^,^18^. Metformin is an oral biguanide anti-diabetictic drug and insulin sensitizer which inhibits glycogenolysis and gluconeogenesis^7^. It also possesses anticancer activity against several tumors through induction of apoptotic signaling and cell cycle arrest^9^. Therefore, the aim of the current study was to investigate the protective effect of metformin against experimentally-induced prostatic hyperplasia in rats with emphasis on role of IGF-1/IGF-1R cascade.

In the current study, treatment of rats with 500 and 1000 mg/kg metformin orally for 14 days significantly inhibited testosterone mediated increase in the prostate weight and prostate weight/body weight ratio compared to the testosterone treated group. Additionally, histological examination confirmed these previous changes in prostate weight and prostate index where co-treatment with metformin (500 and 1000 mg/kg) attenuated the pathological alterations induced by testosterone. Based on these data, metformin at doses 500 & 1000 mg/kg has proven efficacy in protecting against testosterone-induced BPH and further mechanistic investigations were carried out.

Over-expression of AR has been shown in testosterone-treated animals. It is worthy noted that castration significantly decreased, while androgens increased the AR mRNA levels in the prostate^19^. However, in the present study, co-treatment with metformin insignificantly ameliorated testosterone-induced expression of AR and 5 α-reductase II in the prostate tissues. A growing body of evidence suggested the role of estrogen action via its distinct receptors; ER-α and ER-β in the development and progression of BPH^2^. Previous studies demonstrated that testosterone induced ER-α expression but decreased that of ER-β and its downstream effector; P21 in experimental model of BPH^20^. Also, androgen treatment induced prostatic hyperplasia *in-vivo* is dependent on increased expression of growth factors mainly IGF-1 and IGF-1R^21^ and increased expression of phosphorylated Akt as well as Bcl-2^22^.

Estrogen receptors (ERs) regulate cellular proliferation and differentiation, such that high ER-α to ER-β expression ratio is an important determinant of BPH progression^23^. ER-α activation induces proliferative and anti-apoptotic responses, however, ER-β activation served a beneficial role in the prostate through induction of pro-apoptotic cascade^3^. Particularly, ER-α induces the expression of IGF-R, a member of tyrosine kinase receptors, whose activation stimulate several mitogenic and pro-survival cascades mainly (PI3K/Akt)^24^,^25^. Akt phosphorylation and activation may lead to cell death dysregulation through inhibiting the expression of pro-apoptotic proteins as Bax and Bad while provoking the expression of anti-apoptotic proteins as Bcl-2^26^.

The cross talk between ER-α, ER-β and IGF-1R exists such that breast cancer cell lines with suppressed IGF-1R expression showed low level of ER-α but high level of ER-β^27^. ER-β induces cyclin-dependent kinase inhibitor 1A; P21 gene expression resulting in cell cycle arrest and decreased cellular proliferation^28^. In addition, P21 also plays an important role in regulation of apoptosis where overexpression of P21 in human hepatoma cell lines induces the expression of Bax and suppresses the anti-apoptotic effect of Bcl-2 through modulating the ratio of Bcl-2 to Bax^29^.

It has been demonstrated that metformin successfully down-regulated the expression ER-α in MCF-7 cell lines^30^. The inverse relationship between ER-β and ER-α expressions have been previously characterized^31^. Metformin increases p21 and Bax/Bcl-2 expressions^32^, as well as, inhibits IGF-1, IGF-1R and pAkt expressions. Thus, serves to suppress protein synthesis and cellular proliferation *in-vitro*^33^,^34^. These data are in accordance with our results such that co-administration of metformin (500 and 1000 mg/kg, orally) significantly attenuated mRNA expression of ER- α, IGF-1 and IGF-1R as well as the protein expression of pAkt/total Akt induced by testosterone and elevated that of ER-β and P21 to near control level.

Phosphatase and tensin homolog (PTEN) possesses a phosphatase activity and plays an important role in inhibiting cell survival, growth and proliferation mainly through inhibitor of PI3K/Akt signaling pathway^35^. PTEN is considered as one of the most frequently lost tumor suppressors in cancer mainly prostate cancer^36^. It has been reported that ER-α promotes prostate cancer proliferation in PTEN-deficient mice via regulating pro-survival cascades^37^. *In-vitro* studies showed that the anti-proliferative effect of ER-β involves repressing the expression of AKT which involves PTEN up-regulation^38^,^39^. Further, PTEN mRNA expression was shown to be down-regulated in experimentally–induced BPH^40^. Metformin has been shown to induce PTEN expression, possibly via AMPK dependent pathway^41^.

AMPK is a highly conserved energy-sensing serine/threonine kinase which is activated by metabolic stressors^42^. Activation of AMPK modulates insulin signaling downstream of the insulin receptor by negatively regulating the consequences of activation of PI3K/Akt pathway thus controlling protein synthesis and inhibiting cellular proliferation^43^. Metformin inhibited prostate cancer proliferation through abrogating androgen-induced IGF-1R expression which is partially dependant on AMPK activation^44^. In addition, ER- β plays a significant role in AMPK activation^45^, thus further control cellular proliferation via switching off several survival and oncogenic pathways^46^. In this context, our results showed that co-treatment with metformin 500 mg/kg and 1000 mg/kg significantly ameliorated testosterone–induced reduction in PTEN gene expression and AMPKα [PT-172] activity.

In conclusion, the current findings elucidate the effectiveness of metformin in preventing testosterone-induced BPH in rats. These results could be attributed, at least in part, to its ability to enhance expression ratio of ER-β/ER-α, activate AMPK, decrease IGF-1, IGF-1R and pAkt expressions, increase P21, PTEN, Bax/Bcl-2 expressions with a subsequent downstream inhibition of prostate proliferation.

## Materials and Methods

### Drugs and chemicals

Metformin and testosterone were kindly supplied by the Chemical Industries Development Co. (CID), Giza, Egypt. The protein assay detection kit was purchased from BioVision Inc. (Mountain View, CA, USA) and 5′-AMP-activated protein kinase; phosphorylated at Thr-172 of the alpha subunit (AMPKα[pT172]) ELISA kit was purchased from Invitrogen (Camarillo, CA, USA). PureLink® RNA Mini Kit assay kit was purchased and SYBR Green kit were obtained from Invitrogen (Camarillo, CA, USA). High-Capacity cDNA Reverse Transcription Kit was obtained from Applied Biosystems (Foster City, CA, USA). All other chemicals and solvents were of the highest grade commercially available.

### Animals

This study was carried out using male 10-week Sprague–Dawley rats weighing 200–250 g, purchased from Nile Co. for Pharmaceutical and Chemical Industries, Cairo, Egypt. Rats were housed in an air-conditioned atmosphere, at a temperature of 22 ± 2 °C with alternatively 12 hour light and dark cycles. They were kept on a standard diet and water *ad libitum* and acclimated for one week before experimentation.

### Ethics Statement

Animal care and experiments were conducted in accordance with the protocols approved by the Unit of Biomedical Ehics Research Committee, Faculty of Medicine, King Abdulaziz University, following the Institutional Animal Care and Use Committee guidelines.

### Experimental design

Animals were randomly assigned to four groups; eight animals per group and treated five days per week for two weeks as follows; the first group served as control and received distilled water (1 ml/kg) per orally (p.o.) and olive oil (1 ml/kg, subcutaneously (s.c.). The second group received distilled water, p.o. and 3 mg/kg testosterone dissolved in olive oil, s.c. to induce BPH sharing common features with that occurred in human^47^. The third and fourth groups were given 500 and 1000 mg/kg metformin dissolved in distilled water, p.o. and 3 mg/kg testosterone dissolved in olive oil, s.c. The fifth group received 1000 mg/kg metformin dissolved in distilled water, p.o. and olive oil s.c. Rats were sacrificed by decapitation 72 h after the last s.c. injection. Then, prostate tissues were rapidly dissected out and weighed. Sections of the ventral lobes were fixed in 10% neutral buffered formalin and embedded in paraffin for histological and immunohistochemical examinations. The remainder of each prostate was stored at −80 ^o^C and used for further analyses.

### Prostate weight and prostate index

Prostate tissues were harvested and weighed instantly then prostate index was calculated as the ratio of the prostate weight to the total body weight.

### Histopathological examination

Sections from prostate tissues were fixed in 10% neutral buffered formalin and processed for paraffin sections of 4 μm thickness. Sections were collected on glass slides, deparaffinized then stained with haematoxylin and eosin (H&E) for routine histopathological examination.

### Reverse transcriptase–polymerase chain reaction (RT-PCR) analysis of (ER)-α, ER-β, AR, 5 α-reductase II, IGF-1, IGF-1R, PTEN, P21, Bax and Bcl-2gene expressions

Total RNA was isolated using PureLink® RNA Mini Kit assay kit, according the manufacturer protocol. Reverse transcription was carried out using a High-Capacity cDNA Reverse Transcription Kit. Real time PCR was performed using the following primers: Estrogen receptor (ER)-α sense primer, 5′-TCCTTCTAGACCCTTCAGTG AAGCC -3′ and the corresponding antisense primer, 5′-ACATGTCAAA GATCTCCAC CAT GCC-3′, ER-β sense primer, 5′-TTGGTGTGAAGCA AGAT CACTAGAG-3′ and the corresponding antisense primer, 5′-AACAGGGCTGGCACAACTG-3′, Androgen receptors (AR) sense primer, 5′-CAAAGGGTTGGAAGGTGAGA -3′ and the corresponding antisense primer, 5′-GAGCGAGCGGAAAGTTGTAG-3′, 5 α-reductase II sense primer, 5′-ATTTGTGTGGCAGAGAGAGG-3′ and the corresponding antisense primer, 5′-TTGATTGACTGCCTGGATGG-3′, IGF-1 sense primer, 5′-AAAATCAGCAGTCTTCCAAC-3′ and the corresponding antisense primer, 5′-AGATCACAGCTCCGGAAGCA-3′. IGF-1R sense primer, 5′-TCCACCATAGACT GGTCTCT-3′ and the corresponding antisense primer, 5′-ACGAAGCCATCTGAGTC ACT-3′. PTEN sense primer, 5′-CAATGTTCAGTGGCGGAACTT-3′ and the corresponding antisense primer, 5′-GGCAATGGCTGAGGGAACT-3′. P21 sense primer, 5′-CTGGTGATGTCCGACCTGTTC-3′and the corresponding antisense primer, 5′-CTGCTCAGTGGCGAAGTCAAA-3′. Bax sense primer, 5′- GATCAGCTCGGGCAC TTTAG -3′ and the corresponding antisense primer, 5′-TGTTTGCTGATGGCAACTTC -3′. Bcl-2 sense primer, 5′-AGGATTGTGGCCTTCTTTGAGT-3′ and the corresponding antisense primer, 5′- GCCG GTTC AGGTACT CAGT CAT -3′. β-actin was used as reference housekeeping gene with sense primer, 5′-CCCAGCACAATGAAGATCAA GATCAT-3′and the corresponding antisense primer, 5′-ATCTGCTGGAAGGTGGACA GCGA-3′. All the primers were purchased from Invitrogen (Camarillo, CA, USA). Quantitative real-time PCR was performed using SYBR Green and Applied Biosystems Step One Real Time PCR System to evaluate relative gene expressions. After 1  min hot start at 95 °C, samples underwent denaturation at 95 °C for 15 s, annealing at 60 °C for 1min for 45 cycles. Melting curve analysis was performed starting at 60 °C until 95 °C with stepwise temperature elevations. Fluorescent quantitative analysis was carried out with the thermal cycler’s software package to calculate the ΔCt value where the Ct value is the cycle number when the fluorescence curve crossed the baseline value. ΔCt value = target gene Ct value—reference gene Ct value. ΔΔCt = experimental group ΔCt—control group ΔCt. Gene expression in the control group was assigned a value of unity and the degree of gene expression in experimental group was computed as 2^−ΔΔCt^ and referred to as relative quantification (RQ).

### Assessment of AMPKα [pT172]

*AMPKα [pT172] was assessed in* prostate tissues using (AMPKα [pT172]) ELISA kit Invitrogen (Camarillo, CA, USA) according to the manufacturer protocol and the absorbance was read at 540 nm. Results were expressed as Units/mg protein. Protein content was determined according to the commercially available protein assay kit (BioVision Inc., Mountain View, CA, USA).

### Western Blot Analysis for pAkt & Akt

Western blotting analysis was performed to measure protein expression of p-Akt (polyclonal rabbit antibody, BioVision, SF, USA), total Akt (polyclonal rabbit antibody, BioVision, SF, USA) and β-actin (polyclonal mouse antibody, Santa Cruz, CA, USA). Protein lysates were prepared by treating prostate tissues with RIPA lysis buffer (1 × PBS, 1% Nonidet P-40, 0.5% sodium deoxycholate and 0.1% SDS) for 30 min. The lysates were sonicated twice for 20 sec on ice and then centrifuged at 10 000 *×* *g* for 10 min to sediment the particulate material. Sodium dodecyl sulfate-polyacrylamide gel electrophoresis was performed under reducing conditions on 10% polyacrylamide gels. The resolved proteins were transferred to PVDF (Bio-Rad Laboratories, CA, USA) and the membranes were incubated with suitable primary antibodies in blocking solution. After washing with PBS plus 0.5% Tween-20, the membranes were then incubated with the secondary antibody-HRP in blocking solution for 2 h at room temperature. After washing, membrane blots were developed using ECL^TM^ Western blotting detection chemiluminescent substrate and finally exposed to X-ray film.

### Statistical Analysis

Data are presented as mean ± S.D. Multiple comparisons were performed using one-way ANOVA followed by Tukey-Kramer as a post-hoc test. The 0.05 level of probability was used as the criterion for significance. All statistical analyses were performed using Instat software version 3. Graphs were sketched using GraphPad Prism software version 5 (GraphPad Software, Inc., La Jolla, CA, USA).

## Additional Information

**How to cite this article**: Mosli, H. H. *et al*. Metformin Attenuates Testosterone-Induced Prostatic Hyperplasia in Rats: A Pharmacological Perspective. *Sci. Rep*. **5**, 15639; doi: 10.1038/srep15639 (2015).
